# Realising the potential of patient and public involvement to make a difference: what can trial teams do?

**DOI:** 10.1186/1745-6215-16-S2-O93

**Published:** 2015-11-16

**Authors:** Bridget Young, Louise Dudley, Paula Williamson, Carrol Gamble

**Affiliations:** 1 University of Liverpool, Liverpool, UK; 2 University of Birmingham, Birmingham, UK

## Background

Evidence is needed on how to optimise patient and public involvement within trials. We explored trialists' and PPI contributors' accounts of PPI and what influences its impact.

## Methods

We accessed informants through a cohort of UK NIHR Health Technology Assessment programme trials funded between 2006-10. Analysis of transcripts of audio-recorded qualitative interviews was informed by triangulation of informants' accounts and the principles of the constant comparative method.

## Results

We interviewed 21 chief investigators, 10 trial managers and 17 PPI contributors from 28 trials. Over half the informants indicted that PPI had made a difference, influencing either an aspect of a trial, or how trialists felt about a trial. Informants described how PPI's impact depended on the quality of chief investigators' goals and plans for PPI, the quality of relationships between trialists and PPI contributors, and stage at which PPI involvement was implemented. Including PPI contributors in responsive (e.g. advisory groups) and managerial (e.g. trial management groups) roles was thought more likely to achieve impact than oversight roles (e.g. trial steering committees).

## Conclusions

Strategies which include developing PPI goals at an early stage, matching these to the needs of a trial, planning PPI implementation in accordance with these goals, investing in developing good relationships between PPI contributors and trialists, and favouring responsive and managerial roles for contributors in preference to oversight-only roles have the potential to enhance PPI within trials. These features could inform research funders' judgments about the quality of PPI in grant applications for clinical trials.

